# Trends in Food Consumption Patterns of US Infants and Toddlers from Feeding Infants and Toddlers Studies (FITS) in 2002, 2008, 2016

**DOI:** 10.3390/nu11112807

**Published:** 2019-11-17

**Authors:** Emily W. Duffy, Melissa C. Kay, Emma F. Jacquier, Diane Catellier, Joel Hampton, Andrea S. Anater, Mary Story

**Affiliations:** 1 Gillings School of Global Public Health, University of North Carolina, Chapel Hill, NC 27599, USA; 2 Duke Global Health Institute, Duke University, Durham, NC 27110, USA; melissa.kay@duke.edu (M.C.K.); mary.story@duke.edu (M.S.); 3 Nestlé Research, 1000 Lausanne, Switzerland; Emma.Jacquier1@rd.nestle.com; 4 RTI International, Research Triangle Park, NC 27709, USA; dcatellier@rti.org (D.C.); jhampton@rti.org (J.H.); aanater@rti.org (A.S.A.)

**Keywords:** nutritional epidemiology, Feeding Infants and Toddlers Study, food intakes, trends analysis, eating habits, young children

## Abstract

The Feeding Infants and Toddlers Study (FITS) is the largest survey of dietary intake among infants and young children in the United States. Dietary patterns in early childhood are a key component of prevention of diet-related chronic diseases, yet little is known about how food consumption patterns of infants and young children have changed over time. The objective of this study is to examine trends in food and beverage consumption among children ages 6–23.9 months using data from the FITS conducted in 2002, 2008, and 2016. A total of 5963 infants and young children ages 6–23.9 months were included in these analyses. Food consumption data were collected using a multiple-pass 24-h recall by telephone using the Nutrition Data System for Research. Linear trends were assessed using the Wald’s test in a multivariable linear regression model. Positive significant findings include increases in breast milk consumption and decreases in the consumption of sweets, sugar-sweetened beverages, and 100% fruit juice. More troubling findings include decreasing infant cereal consumption, stagnant or decreasing whole grain consumption, and stagnant consumption of vegetables. Our findings suggest some promising improvements in dietary intake among infants and toddlers in the United States over the past 15 years, but further policy, programmatic, and industry efforts are still needed.

## 1. Introduction

Understanding dietary exposures during different life stages is particularly important among infants and toddlers. Infancy and early childhood are critical periods for the development of taste preferences and dietary patterns [1,2,3]. In addition, early dietary behaviors lay the foundation for future dietary patterns with the potential to prevent diet-related chronic disease in later life [4,5,6]. Understanding how infant and young children’s intake aligns with current dietary guidance is of public health importance. Knowledge of trends in dietary intake patterns of infants and young children in the United States can inform policy and programmatic efforts to improve infant and child health. Yet data on patterns of food and beverage intake are limited for this age group.

Two studies, each using data from the National Health and Nutrition Examination Survey (NHANES), examined trends in food and beverage intake in this age group [7,8]. However, these studies lack recent data and there is limited understanding of population-level changes in food consumption behaviors of young children over time [8]. A more recent analysis using data from the Feeding Infants and Toddlers Study (FITS) examined trends in nutrient intakes among 0–47.9-month-olds and found that nutrient intakes were generally stable from 2002 to 2016; however, sodium intakes exceed recommended levels and there were notable declines in vitamins D and E and fiber among the entire sample and in iron among infants [9].

It is important to examine trends in food consumption alongside trends in nutrient intakes to understand how food consumption behavior may be contributing to observed changes in nutrient intakes. Given the gaps in the literature, the aim of this study is to assess trends in food and beverage consumption (per capita and per consumer) among children ages 6–23.9 months from the FITS conducted in 2002, 2008, and 2016. This study provides the most recent information on young children’s dietary intake and how intake has changed across three time periods in the past 15 years. This information can inform policy, program, practice, and intervention efforts aimed at improving the diets and overall health of infants and young children in the United States.

## 2. Methods

### 2.1. Sample

The FITS is a series of cross-sectional surveys of food and beverage intake in national samples of infants and young children. The 2002 sample was restricted to infants and toddlers 4 months to 24 months old, while the 2008 and 2016 samples include children from birth up to 48 months old. The sampling plans reflect the geographic and sociodemographic diversity of households with young children across the 50 states and Washington DC. The 2002 and 2008 samples were drawn from targeted lists of prenatal and postnatal records from the New Parent Database from Experian Information Solutions, Inc. (Costa Mesa, CA, USA), and the 2016 sample was drawn from a combination of the New Parent Database, address-based and cell phone sampling frames, and a web panel. For all surveys, sampling weights were created for analysis to account for unequal probabilities of selection (resulting from the sample design and planned oversampling of subgroups) and were adjusted for nonresponse. Further details of the sampling and methodology are available in methods publications from each FITS [10,11,12].

A total of 5963 infants and young children ages 6–23.9 months were included in these analyses: 2035 from FITS 2016, 1443 from FITS 2008 and 2495 from FITS 2002. Children less than 6 months old were not included because their diets tend to have limited variability. To permit alignment of ages across the survey years, we further restricted our sample to children ≤23.9 months.

### 2.2. Data Collection

Structured telephone interviews were conducted by highly trained interviewers with the primary caregiver of the selected child (one child per household) in English or Spanish. Age-specific recruitment and feeding practices questionnaires were used to assessed household demographics, characteristics of the child, feeding practices, and eating behaviors, with separate questionnaires for infants and young children below 24 months of age and for children 24 months and older. Following the recruitment and feeding practices questionnaires, a multiple-pass 24-h recall was conducted by telephone using the Nutrition Data System for Research (NDSR; University of Minnesota, Minneapolis, MN, USA). A second 24-h recall was collected at least one week later on a random subset of respondents (23% in 2002 [10], 21% in 2008 [11], and 25% in 2016 [12]) to estimate usual nutrient intakes.

### 2.3. Data Analysis

Statistical analyses were performed using SAS (version 9, SAS Institute Inc., Cary, NC, USA) and SAS-callable SUDAAN^®^ (version 11, RTI International, Research Triangle Park, NC, USA) software. Except for descriptive statistics of the sample participants (which were unweighted; Table 1), all analyses used the sampling weights to account for differential probabilities of selection and the complex sample design.

Breast milk volumes for breastfed infants were estimated consistently across the 2002, 2008, and 2016 surveys using procedures developed for FITS [13,14,15] and adopted by the Centers for Disease Control and Prevention for the NHANES [16]. Volumes of 600 mL, 89 mL, or 59 mL per feeding were assigned for breastfeeding infants 6–11.9 mo, 12–17.9 mo, or 18–23.9 mo old, respectively [11].

The infant formula food category includes ready-to-feed, concentrated, or dry powder formulations. For concentrated or dry powder formula, grams of infant formula was calculated as reconstituted values (i.e., grams of concentrated/dry formula and grams of water combined) [17]. Across the FITS years, quantities of water were determined according to standard/recommended preparation instructions or by the respondent reporting. In FITS 2016, field interviewer training emphasized collection of preparation instructions whenever possible, even if the preparation was described by the respondent as standard. (Reported grams of infant formula per consumer and per capita may differ from those published previously [18], as those estimates did not consider reconstituted formulations of concentrated/formula).

The percentage of children consuming specific foods on the day of the survey and the amounts of foods consumed by those individuals (mean grams per consumer) were calculated. Per capita consumption data (mean grams) is also presented to provide an estimation of food intake behaviors at a population level, including consumers and non-consumers of each food. Linear trends from 2002 to 2016 in food consumption parameters (percent consuming, grams consumed per capita and per consumer) were assessed using the Wald test in multivariable logistic and linear regression models. All models were stratified by age (6–11.9 mo, 12–23.9 mo) Following the rationale recently described by Benjamin et al. to reduce the rate of false positive findings [19], we considered a value of *p* < 0.001 (rather than *p* < 0.05) statistically significant, and suppressed *p*-values based on fewer than 30 observations (which may be 30 consumers or 30 non-consumers). Suppressed *p*-values are denoted in tables as NA and footnoted.

## 3. Results

### 3.1. Demographics

The samples were mostly non-Hispanic white and married (Table 1). Between 24% and 35% of the samples had household incomes less than 100% of the federal poverty level. Participation in the Special Supplemental Nutrition Program for Women, Infants, and Children (WIC) ranged from 27% to 31%. More than half of the sample had a college- or graduate-level education. There were some notable demographic differences in the FITS samples across the three surveys, such as a higher percentage of non-Hispanic Black children in the 2016 sample compared to 2002 and 2008, and a higher percentage of respondents with some secondary education in 2016 compared to 2002 and 2008.

### 3.2. Any Milk and Milk Products

6–11.9-month-olds. All infants consumed milk or milk products (includes breast milk and/or infant formula) in all three survey years. The amount of milk and milk products consumed per capita remained stable over time with 6–11.9-month-olds consuming, on average, 755 g of milk and milk products per capita. The major sources of milk were formula and breast milk; about 10% of infants aged 6–11.9 months consumed cow’s milk in all three years.

12–23.9-month-olds. Almost all (99%) 12–23.9-month-olds consumed milk and milk products with only 1% not consuming any milk products on the day of the survey, across the three time periods. There were no changes over time in the percent of children consuming or the amount consumed per capita. Cow’s milk followed by infant formula and cheese were the most prevalently consumed milk products.

#### 3.2.1. Breast Milk and Infant Formula

6–11.9-month-olds. Among 6–11.9-month-olds, there was a significant increase in the percent of infants consuming breast milk on the day of the survey. This was coupled with a significant decrease in the percent of infants consuming infant formula (Table 2). Among consumers of infant formula (Table S1), there was a significant increase in the grams of infant formula consumed.

12–23.9-month-olds. Among 12–23.9-month-olds, there were no significant changes in the percent of infants consuming breast milk from 2002 to 2008 to 2016, but the percent consuming infant formula did decrease (Table 2). There was a significant decrease in the grams of infant formula consumed per capita but no significant difference per consumer (Table S1).

#### 3.2.2. Cow’s Milk

6–11.9-month-olds. Approximately 10% of 6–11.9-month-olds consumed cow’s milk in all three FITS surveys. This prevalence remained stable over the 3 survey years. Small sample sizes limited our ability to assess changes in cow’s milk varieties (whole, reduced fat, low- or nonfat) other than the general category of cow’s milk. There were no significant changes in the amount of cow’s milk consumed per capita among survey years; 6–11.9-month-olds consumed on average 31 g of cow’s milk per capita on the day of the survey (Table 2). However, among those consuming cow’s milk, infants consumed on average 348 g per consumer, which is approximately 1.4 cups, with the majority coming from whole milk, followed by reduced-fat (2%) milk (Table S1).

12–23.9-month-olds. There were also no significant changes in the percent of 12–23.9-month-olds consuming cow’s milk across survey years, with approximately 85% of children consuming cow’s milk on the day of the survey. There was a significant decrease in grams per capita of cow’s milk. The percent of 12–23.9-month-olds consuming reduced fat (2%) cow’s milk decreased across the three study periods (Table 2). The amount of reduced fat (2%) cow’s milk consumed per capita also decreased significantly. There were no significant differences in the percent of toddlers consuming or the amount consumed per capita for any other subcategory of cow’s milk (whole, low-fat, nonfat, or flavored). However, on a per consumer basis, there was a significant decrease in the grams of whole milk consumed (Table S1). Whole milk was the most commonly consumed variety of cow’s milk among 12–23.9-month-olds, and remained stable with about two-thirds (64%) consuming whole milk on the day of the survey.

#### 3.2.3. Cheese and Yogurt

6–11.9-month-olds. There were no significant changes in the percent of 6–11.9-month-olds consuming cheese or yogurt or in the grams per capita consumed (Table 2). Less than 10% of 6–11.9-month-olds consumed cheese or yogurt over the three survey years.

12–23.9-month-olds. For 12–23.9-month-olds, 30% to 41% consumed cheese on the day of the survey and there were no changes in the percent of children consuming cheese or the amount of cheese consumed per capita (Table 2). There was a significant increase in the percent of 12–23.9-month-olds consuming yogurt and in the grams of yogurt consumed per capita. Among toddlers who did consume yogurt on the day of the survey, the amount remained stable at about 116 g per consumer (Table S1).

### 3.3. Grains and Grain Products

6–11.9-month-olds. There was a significant decrease in the percent of 6–11.9-month-olds consuming any grains or grain products across the three survey years (Table 3). There was a significant decrease in grams consumed per capita for all grain products, but no change in grams per consumer (Table S1). Between 2008 and 2016, there was a decrease, though not statistically significant, in the percent of 6–11.9-month-olds consuming any whole grains (whole grain intake was not assessed in the 2002 FITS). There were no significant changes in the grams of any whole grains consumed per capita between these two time periods.

The percent of infants 6–11.9 months old consuming infant cereal decreased significantly, as did the grams per capita (Table 3). However, on a per consumer basis, the grams of infant cereal consumed did not change, with an average of 44 g (Table S1). Family cereal consumption remained stable with approximately 20% of infants consuming family cereal on the day of the survey; however, there were no significant changes in amount consumed, either per capita or per consumer.

The percent of infants consuming grain-based baby finger foods increased significantly over time, as did the grams consumed per capita (Table 3). Average grams per consumer was higher at 6 g, but there was no significant change across survey years (Table S1).

12–23.9-month-olds. There were no changes in the percent of toddlers consuming any grain or grain products (approximately 96% consuming) nor in the grams per capita. There was no significant change between 2008 and 2016 in the percent of children consuming any whole grains. Between 64 and 69% of 12–23.9-month-olds consumed any whole grains on the day of the survey. However, there was a significant increase in the amount of any whole grains consumed per capita and per consumer.

There were no changes in the percent consuming or the amount consumed of infant cereal, or family cereal, consumed over the three survey years. On a per consumer basis, 12–23.9 months olds had on average 49 g of infant cereal and 52 g of family cereal (Table S1). Family cereal was the most commonly consumed grain type, consumed by approximately 50%–60% of toddlers, followed in popularity by bread and then crackers.

### 3.4. Fruit and 100% Fruit Juice

6–11.9-month-olds. There was a slight, non-significant decline in the percent of 6–11.9-month-olds consuming all fruits (including 100% fruit juice); However, the grams consumed per capita did decrease significantly (Table 4). There was a significant decline over the three FITS surveys in percent of 6–11.9-month-olds consuming 100% fruit juice and the amount of 100% fruit juice consumed per capita. However, there was no change in the grams per consumer for either all fruits (including 100% fruit juice) or 100% fruit juice (Table S1). There were no changes in the percent consuming any fruit (excluding 100% juice), which remained stable at approximately 74%, or the amount consumed.

12–23.9-month-olds. There was no change in the percent of 12–23.9-month-olds consuming any fruit (including 100% fruit juice) nor in the grams per capita (Table 4). Among 12–23.9-month-olds there was a significant decrease in the percent of children consuming any 100% fruit juice and the amount of 100% fruit juice consumed per capita. However, there was no change in the grams per consumer of 100% fruit juice, with an average of 244 g (Table S1). There was a statistically significant increase in the amount of whole fruit consumed per capita, while the percent of children consuming whole fruit increased somewhat in 2016 (Table 4), as did the grams per consumer (Table S1), neither of these changes was statistically significant.

### 3.5. Vegetables

6–11.9-month-olds. While the percent of infants consuming any vegetable increased slightly over the three survey years, the change was not significant (Table 5). Between 28% and 33% of 6–11.9-month-olds did not consume a distinct vegetable on the day of the survey. Baby food vegetables were the most commonly consumed type of vegetables over the three surveys, with an average of 44% of infants consuming in 2002, 2008, and 2016. The only significant changes observed in vegetable intake over the three FITS were increases in the percent of 6–11.9-month-olds consuming non-baby food vegetables and the amount of non-baby food vegetables consumed. However, there was no change in the grams per consumer of non-baby-food vegetables (Table S1). Non-baby food vegetables comprised about 20% of total vegetable consumption, and there were no changes in the percent of children consuming the overall category of vegetables or in the amount consumed.

12–23.9-month-olds. There were no significant changes in the percent of children consuming any vegetable or in the amount consumed per capita or per consumer as measured by the three FITS (Table 5). Between 21% and 28% of 12–24-month-olds did not consume any vegetables on the day of the survey.

### 3.6. Meat or Other Protein Food

6–11.9-month-olds. There was a significant increase, over the three surveys, in the percent of infants consuming any meat or other protein food, as well as the percent consuming non-baby food meats and other protein sources (Table 6). Not enough participants consumed baby food meats (<5%) to evaluate changes. Significant changes also were observed in the amounts of these food groups consumed per capita. There was no change in grams per consumer for any meat or other protein group (Table S1).

12–23.9-month-olds. Among children 12–23.9 months old, there was no change in the percent of children consuming the overall category of any meat or other protein food, but there was a significant increase in the amount of this food category consumed per capita (Table 6) and per consumer (Table S1). There were no changes in non-baby food meats (even fewer toddlers consumed baby food meats than infants, so changes could not be evaluated). However, over time, the percent of children consuming other protein sources (e.g., nuts, beans, seeds) and the amount of these other protein sources consumed per capita increased significantly, but amounts did not significantly change per consumer (Table S1).

### 3.7. Sweets, Sweetened Beverages, Desserts, and Savory snacks

6–11.9-month-olds. There was a decrease in both the percent of infants consuming the overall category of all sweets, sweetened beverages, and desserts and in the amount consumed per capita (Table 7) and per consumer (Table S1). There were similar decreases in the percent consuming sweets (i.e., excluding sugar-sweetened beverages) and in amount of sweets consumed per capita and per consumer (Table S1). It was not possible to conduct statistical tests on the category of sugar-sweetened beverages (SSBs), or specific types of SSBs, for 6–11.9-month-olds, given the small sample size of consumers: less than 10% of 6–11.9-month-olds consumed SSBs in all survey years. Fruit-flavored drinks were the most commonly consumed type in all survey years with about 6% of 6–11.9-month-olds consuming them.

12–23.9-month-olds. Similarly, among 12–23.9-month-olds, there were decreases in the percent of children consuming and amounts consumed for the categories of all sweets and SSBs (Table 7). There was a significant decrease in percent of children consuming and grams per capita for any sweets. There was also a significant decrease over the three surveys in the percent of children consuming any SSBs or fruit-flavored drinks, and in the amounts of these beverages consumed per capita (Table 7), but not per consumer (Table S1). It was not possible to test changes in sports drinks or soda consumption, because few toddlers consume these. There were no significant changes in savory snack consumption over time.

## 4. Discussion

Using cross-sectional data from three different FITS (2002, 2008, and 2016), we identified some positive shifts in the diets of infants and young children in the United States, including increases in breastfeeding prevalence and decreases in consumption of 100% juice, sweets, SSBs, and desserts; but we also identified concerning trends such as decreases in the percent of infants consuming whole grains and stagnation in vegetable intake. Our results can be used to guide the efforts of health care providers, public health practitioners, researchers, and policymakers to improve the diets and overall health of infants and young children.

A key finding was the increase in prevalence of breast milk consumption among 6–11.9-month-olds over the three survey years. Breastfeeding is recommended by the American Academy of Pediatrics (AAP) until 1 year of age and longer if mutually desired by the mother and the infant [20]. A recent trend analysis of NHANES 2005–2008 and 2009–2012 data found no changes over time in the percent of 6–12-month-olds breastfeeding [7]. However, another NHANES analysis encompassing a longer time period compared 1997–2000 and 2009–2012 breastfeeding prevalence and found the percent of children still breastfeeding at 6 months increased significantly from 36% to 43% [21]. Several secular trends may explain the observed increase in breastfeeding prevalence. These include changes in WIC policies and programs, including implementation of a breastfeeding peer counseling support program in 2004 and revisions to the WIC food package in 2009 to better support breastfeeding. WIC is a federal nutrition assistance program for low-income pregnant, post-partum, and breastfeeding women, infants, and children up to age 5 that provides nutritious foods, nutrition education, breastfeeding support and health care referrals. Other trends that may have contributed to increases in breastfeeding prevalence include increases in the number of baby friendly hospitals; improved workplace support for breastfeeding mothers; increased insurance coverage for breast pumps; and decreases in infant formula advertising spending [22,23,24]. While these are promising trends, future efforts should emphasize ensuring that these positive changes are realized equally among all sociodemographic groups.

There were few shifts in consumption of cow’s milk or other dairy products. A concern noted across all survey years was that approximately 10% of 6–11.9-month-olds were consuming cow’s milk, which is not recommended by the AAP or other expert groups until 1 year of age [20,25]. We observed a significant increase in yogurt consumption among 12–24.9-month-olds. Although evidence suggests yogurt can contribute key nutrients, like calcium, to children’s intake, products vary widely in sugar content, and yogurt positioned for consumption by children may be higher in added sugars [26,27].

Over the last decade, there has been notable reformulation of grain-based products to increase whole grain content; labeling of whole grain foods has also improved in that time frame. Despite that, we observed declines in the percentage of 6–11.9-month-olds consuming whole grains, and in the amount consumed per capita. There was no change in the percent of 12–23.9-month-olds consuming whole grains, but an increase in the amount of whole grains consumed, perhaps due to reformulation. Studies using NHANES data to examine trends in the amounts of whole grains consumed among older children and adolescents have found mixed results, with intake either improving or staying the same over time [28,29,30]. There is still a notable percentage of infants and young children who did not consume whole grains on the day of the survey. This should be a focus of future policy and programmatic efforts because whole grains are an important source of fiber, which is an under-consumed nutrient among young children in the United States [31].

Among 6–11.9-month-olds, there was a decrease in the consumption of infant cereal over the three survey years. Iron-fortified infant foods can be a key source of much-needed iron during this life stage, and about 20% of 6–11.9-month-olds are at risk of inadequate iron intake [31,32]. Data from the FITS 2016 suggest that infant cereal and meats are some of the most commonly consumed complementary foods among 6–11.9-month-olds, behind fruits and vegetables [33]. Moreover, some FITS data indicate that the types of meats consumed are usually poultry or cold-cuts, which often contain less bioavailable sources of iron than beef [34]. However, in 2013, the AAP released guidance for parents to offer a wide variety of foods to decrease exposure to arsenic in rice and rice products in response to a U.S. Food and Drug Administration (FDA) risk assessment. The FDA recommended to continue to feed infant cereal made from other grain sources [35]. This guidance and the FDA risk assessment may have contributed to declines in consumption. If the decline of infant cereal, a key source of iron for infants, is not replaced with other bioavailable sources of iron in the diet, lower consumption of infant cereal could contribute to inadequate iron intakes. Efforts should be made to help parents and caregivers understand the importance of adequate iron consumption in infancy and appropriate sources of iron, such as infant cereals made from oats, wheat, and barley, as well as meats.

There was no significant change in the percent of children consuming any distinct portion of vegetables or the amount of any distinct portion of vegetables consumed over the three time points. Still, about one in four children from 6–23.9 months are not consuming any vegetables on a given day. There was a slight increase among 6–11.9-month-olds in consumption of non-baby food vegetables, perhaps attributable to the rise of the baby-led weaning infant feeding style, which encourages self-feeding of family foods in whole form [36]. Establishing preferences for vegetables early in life is critical for continued consumption throughout early childhood [2,37]. Similar to our findings, a trend analysis of NHANES data from 2003–2010 found no changes in vegetable intake for children 2–18 years old, with no children meeting the Healthy People 2020 vegetable intake targets [38]. Vegetables are a critical source of fiber, vitamins, and minerals throughout life [30]. Infants may initially reject vegetables due to their relatively bitter taste profile; however, there are many strategies that parents and caregivers can employ, such as repeated exposure, pairing vegetables with liked foods, and modeling healthy eating, that can improve acceptability and consumption. In addition to these individual-level strategies, approaches at all levels of the socioecological model are drastically needed to improve vegetable intake in infancy and early childhood [39].

We found declines in the percent of children consuming 100% fruit juice and the amount of 100% fruit juice consumed. The AAP recommends that no 100% fruit juice be consumed prior to 1 year and that 1–3 year olds should consume whole fruit if possible, but if not, a maximum of 4 ounces of 100% fruit juice per day is recommended to meet fruit intake recommendations [40]. These AAP recommendations were released in 2017, so were not a contributor to the observed declines. However, prior to these recommendations, there was some evidence suggesting that 100% fruit juice intake in early childhood may contribute to excess energy intake, increased risk of dental caries, and a heightened preference for sweet foods and beverages [41,42,43]. Additionally, in 2009, the WIC food package was updated, no longer allowing 100% fruit juice purchases for infants and reducing the amount of 100% fruit juice included for 1–5-year-old children. Approximately 30% of the FITS sample participated in WIC at the time of the survey, so it is plausible these policy changes impacted intake. Similar declines in 100% fruit juice among this age group have been observed in other studies [7,44]. There were no significant shifts in whole fruit consumption across survey years. Our findings differ from an analysis of NHANES data from children ages 2 to 18 that found increases in whole fruit consumption between 2003 and 2010 [38].

Unlike fruits, vegetables, and whole grains, most children in the age groups included in this study meet the intake recommendations for total protein [25,31,45]. We observed increases in protein consumption over the three surveys. The increase in non-meat protein consumption found among 12–23.9-month-olds is important, as some varieties of meat can be a key source of essential micronutrients such as B12 and bioavailable iron. Similar increases in non-meat protein sources in this age group have not been observed in trend analyses of NHANES data; however, those analyses include data only up to 2012 [7].

The trends we observed in consumption of sweets and SSBs are encouraging. Yet, there are still many infants and young children consuming these foods and beverages despite recommendations that no added sugar should be consumed prior to 2 years [25,46]. In trend analyses using NHANES data among this age group, similar declines have been observed in sweet and dessert consumption, but not in SSB consumption. In older age groups (2 to 18 years), NHANES data shows similar declines in SSB consumption over time [47]. The most commonly consumed variety of SSB in this age group was fruit flavored drinks at all three time points, making these beverages an important target for future intervention efforts.

A key limitation of this study is the use of parent or caregiver reported intake, which is inherently impacted by measurement error and some degree of reporting bias. Additionally, we did not have sufficient sample size to examine differences in trends by racial/ethnic group. This will be an important area to examine in future research to understand if observed improvements in dietary intake are realized equally among all infants and toddlers. There were many strengths to this study. This is, to our knowledge, the most comprehensive evaluation of trends in dietary intake in this age group. The FITS is one of the largest national studies that examines the dietary intake of infants and young children and utilizes rigorous study design and state-of-the-art 24-h dietary recall methodology.

## 5. Conclusions

Our findings suggest some promising improvements in dietary intake among infants and toddlers in the United States over the past 15 years, but there are still many areas where further policy, programmatic, and industry efforts are needed. While our findings are not causal, it is plausible that federal nutrition program policy changes, organizational policy changes, and industry reformulation efforts over the last decade have supported these improvements in intake. However, more work is needed to bring young children’s dietary intake in alignment with recommendations. Future efforts should examine whether changes in intake observed have occurred across all sociodemographic groups. Unfortunately, other recent studies show that the most vulnerable populations often have the smallest improvements in dietary intakes or diet-related health outcomes [48,49]. Findings from this study can be used to identify critical targets for interventions that will be needed at all levels of the socioecological model to improve intake in this critical life stage.

## Figures and Tables

**Table 1:** Demographic information for the three The Feeding Infants and Toddlers Study (FITS).

Characteristic	% Reporting			*p*-Value		
	2002	2008	2016	2002 vs. 2008	2002 vs. 2016	2008 vs. 2016
***n***	2495	1433	2035			
**Age (months)**						
6–11.9	59	36	44	**<0.0001**	**<0.0001**	**<0.0001**
12–23.9	41	64	56	**<0.0001**	**<0.0001**	**<0.0001**
**Household Income (% of FPL)**						
0%–49%	21	16	16	**0.0006**	**<0.0001**	0.8169
50%–99%	14	8.4	15	**<0.0001**	0.2809	**<0.0001**
100%–199%	38	23	32	**<0.0001**	**<0.0001**	**<0.0001**
200%–299%	19	23	20	0.0141	0.4192	0.0952
300% or above	7.3	30	16	**<0.0001**	**<0.0001**	**<0.0001**
**WIC Participant**	31	27	28	0.5982	**<0.0001**	**<0.0001**
**Marital Status**						
Married	83	82	71	0.4919	**<0.0001**	**<0.0001**
Separated/Divorced/Widowed	2.8	3.2	5.5	0.4971	**<0.0001**	**0.0008**
Never Married/Living with Partner	14	15	23	0.6934	**<0.0001**	**<0.0001**
**Child’s Race/Ethnicity**						
Hispanic	12	11	14	0.2004	0.0549	0.0013
Non-Hispanic White	79	74	68	**<0.0001**	**<0.0001**	**<0.0001**
Non-Hispanic Black	7.4	7.8	13	0.5734	**<0.0001**	**<0.0001**
Non-Hispanic Other	1.7	7.4	4.6	**<0.0001**	**<0.0001**	**0.0001**
**Child ever breastfed**	75.44	80	83	0.0151	**0.0000**	0.0343
**Child low birth weight (<2500 g)**	8.8	12	8.6	**0.0003**	0.8521	**0.0003**
**Respondent Education**						
High school or less	32	25	22	**<0.0001**	**<0.0001**	0.0252
Some post-secondary	15	19	23	0.0081	**<0.0001**	**0.0005**
College or graduate work	53	56	55	0.0464	0.2710	0.3389

Bolded *p*-values are statistically significant at a value of *p* < 0.001.

**Table 2:** Percent consuming and grams per capita consumed of milk and milk products.

Foods	Percent Consuming				Grams Per Capita			
	2002	2008	2016	*p*-Value Linear Trend	2002	2008	2016	*p*-Value Linear Trend
	**6–11.9 months**							
**Any Milk or Milk Products ^1^**	100	100	100	NA	727	734	805	NA
Breast milk	25	34	40	**<0.0001**	149	193	224	**<0.0001**
Formula	80	70	65	**<0.0001**	536	502	531	0.8875
Milk (any) ^2^	8.5	9.5	11	0.1905	33	28	38	0.6106
Cow’s milk ^3^	7.9	9.1	10	0.1751	30	27	36	0.5023
Whole milk ^4^	6.0	6.7	5.8	NA	25	22	27	NA
Reduced fat (2%) milk ^4^	1.7	2.1	3.6	NA	5	5	8	NA
Low- or Nonfat milk ^4^	0.3	0.4	1.0	NA	<0.5	<0.5	1	NA
Flavored milk ^5^	0.4	0.03	0.3	NA	1	<0.5	<0.5	NA
Toddler milk	0.1	0	0.5	NA	<0.5	0	1	NA
**Cheese**	7.0	8.0	9.0	0.1466	2	2	2	0.6552
**Yogurt**	7.9	4.8	7.7	0.9453	7	5	7	0.8320
	**12–23.9 months**							
**Any Milk or Milk Products ^1^**	98	98	98	NA	599	519	504	NA
Breast milk	7.9	6.2	12	0.0035	34	18	38	0.4443
Formula	9.5	9.7	4.2	**<0.0001**	44	49	21	**<0.0001**
Milk (any) ^2^	87	86	87	0.9656	486	421	400	**<0.0001**
Cow’s milk ^3^	84	83	83	0.4495	466	404	383	**<0.0001**
Whole milk ^4^	64	60	67	0.1342	343	295	315	0.1589
Reduced fat (2%) milk ^4^	19	23	13	**0.0005**	103	94	47	**<0.0001**
Low- or Nonfat milk ^4^	5.6	3.7	7.1	0.2141	20	15	21	0.8208
Flavored milk ^5^	3.4	7.0	5.5	0.0925	11	21	14	0.5606
Toddler milk	0.1	0.04	1.2	NA	<0.5	<0.5	2	NA
**Cheese**	41	30	37	0.2049	12	9	10	0.0137
**Yogurt**	17	13	24	**0.0002**	20	15	29	**0.0007**

Bolded *p*-values are statistically significant at a value of *p* < 0.001. NA = *p*-value suppressed because it was based on an insufficient number of observations (fewer than 30 consumers or fewer than 30 non-consumers). ^1^ Excludes dairy products in mixed dishes. ^2^ Includes all milks except breastmilk and infant formula. ^3^ Includes all fat levels, as well as flavored, unflavored, or powdered. ^4^ Includes only unflavored cow’s milk of specified fat level, excludes flavored and powdered. ^5^ Includes flavored cow’s milk, flavored plant milks, and flavored dairy substitutes.

**Table 3:** Percent consuming and grams per capita consumed of grains and grain products.

Food	Percent Consuming				Grams Per Capita			
	2002	2008	2016	*p*-Value Linear Trend	2002	2008	2016	*p*-Value Linear Trend
	**6–11.9 months**							
**All grains and grain products ^1^**	91	90	84	**<0.0001**	56	53	42	**0.0001**
Bread, rolls, biscuits, bagels, and tortillas	12	8.3	12	0.7554	3	2	2	0.7345
Bread	9.2	7.7	9.5	0.8130	2	2	2	0.9591
Crackers, pretzels, and rice cakes	14	11	7.0	**<0.0001**	1	1	1	0.0327
Crackers	13	10	6.1	**<0.0001**	1	1	1	0.0237
Pancakes, waffles, and French toast	3.3	4.0	4.1	NA	1	1	1	NA
Pancakes	1.8	2.8	1.9	NA	1	1	1	NA
Pasta and rice	9.3	11	12	0.1665	5	10	6	0.4570
Pasta	5.1	3.3	4.4	NA	3	1	2	NA
Infant cereal ^2^	76	65	52	**<0.0001**	41	23	22	**<0.0001**
Family cereal ^3^	23	23	19	0.1286	5	13	8	0.1133
Baby finger foods ^4^	6.7	23	33	**<0.0001**	<0.5	1	2	**<0.0001**
**Whole grains (any) ^5^**	—	81	70	0.0024	—	37	30	0.3733
	**12–23.9 months**							
**All grains and grain products ^1^**	97	96	96	0.1349	89	85	95	0.1676
Bread, rolls, biscuits, bagels, and tortillas	52	45	43	0.0015	14	14	16	0.2552
Bread	43	34	35	0.0036	11	10	11	0.5653
Crackers, pretzels, and rice cakes	42	37	36	0.0274	6	6	8	0.1135
Crackers	37	33	33	0.1718	5	6	7	0.0414
Pancakes, waffles, and French toast	15	15	17	0.2595	8	6	9	0.2369
Pancakes	6.3	6.1	8.9	0.0472	4	2	5	0.1081
Pasta and rice	35	30	28	0.0047	28	21	22	0.0768
Pasta	22	12	7.6	**<0.0001**	18	8	7	**<0.0001**
Infant cereal ^2^	11	9.8	11	0.6941	5	4	6	0.4548
Family cereal ^3^	57	61	53	0.1170	25	32	32	0.0367
Baby finger foods ^4^	6.6	9.8	11	0.0011	1	1	1	0.3007
**Whole grains (any) ^5^**	—	65	69	0.1798	—	34	50	**0.0003**

Bolded *p*-values are statistically significant at a value of *p* < 0.001. NA = *p*-value suppressed because it was based on an insufficient number of observations (fewer than 30 consumers or fewer than 30 non-consumers). — = category not assessed for that FITS year. ^1^ Excludes grains in mixed dishes. ^2^ Includes any kind of baby-food cereal, regardless of grain (i.e., rice, oat, quinoa, wheat, multigrain, or unknown grain). ^3^ Includes any ready-to-eat or hot cereal that is not infant cereal. ^4^ Includes pretzels, crackers, rice cakes, and baby-food puffs. ^5^ Includes products that are ≥50% whole grain. Whole grain intake was not assessed in 2002.

**Table 4:** Percent consuming and grams per capita consumed of fruit and 100% fruit juice.

Food	Percent Consuming				Grams Per Capita			
	2002	2008	2016	*p*-Value Linear Trend	2002	2008	2016	*p*-Value Linear Trend
	**6–11.9 months**							
**Any Fruit or 100% Fruit Juice ^1^**	83	83	79	0.1953	163	129	133	**0.0005**
100% juice ^2^	43	30	24	**<0.0001**	69	40	39	**<0.0001**
All fruits (excluding 100% fruit juice) ^3^	73	74	75	0.3302	94	89	94	0.9297
Baby food fruit ^4^	58	47	50	0.0015	72	54	59	0.0077
	**12–23.9 months**							
**Any Fruit or 100% Fruit Juice ^1^**	89	90	90	0.5139	266	254	238	0.0196
100% juice ^2^	60	57	48	**<0.0001**	156	145	108	**<0.0001**
All fruits (excluding 100% fruit juice) ^3^	75	75	81	0.0088	110	109	131	**0.0010**
Baby food fruit ^4^	10	8.2	9.8	0.9707	12	9	12	0.9742

Bolded *p*-values are statistically significant at a value of *p* < 0.001. ^1^ Includes fruit, 100% fruit juice, and baby food fruit/100% juice. Beverages that are <100% fruit juice are included in sugar sweetened beverages. Excludes fruits in mixed dishes. ^2^ Includes any 100% fruit juice regardless of whether it is specifically labeled for babies or not. ^3^ Includes commercial and homemade pureed baby-food fruit and non-baby-food fruit; excludes 100% juice. ^4^ Includes commercial and homemade pureed baby-food fruit, excludes 100% juice.

**Table 5:** Percent consuming and grams per capita consumed of vegetables.

Food	Percent Consuming				Grams Per Capita			
	2002	2008	2016	*p*-Value Linear Trend	2002	2008	2016	*p*-Value Linear Trend
	**6–11.9 months**							
**Any vegetable ^1^**	67	66	72	0.0238	69	73	75	0.1506
Baby-food vegetables ^2^	48	39	45	0.3813	54	46	51	0.5320
Non baby-food vegetables ^3^	17	27	29	**<0.0001**	9	21	17	**0.0003**
White potatoes ^4^	14	11	14	0.6907	5	7	7	0.1676
Fried potatoes ^5^	4.9	3.1	4.2	NA	1	1	1	NA
	**12–23.9 months**							
**Any vegetable ^1^**	79	73	72	0.0061	69	65	64	0.2216
Baby-food vegetables ^2^	8.2	7.3	5.7	0.0280	10	10	6	0.0040
Non baby-food vegetables ^3^	61	54	60	0.6331	38	36	39	0.7325
White potatoes ^4^	36	32	30	0.0238	21	19	19	0.4040
Fried potatoes ^5^	18	14	12	0.0028	8	5	5	0.0332

Bolded *p*-values are statistically significant at a value of *p* < 0.001. NA = *p*-value suppressed because it was based on an insufficient number of observations (fewer than 30 consumers or fewer than 30 non-consumers). ^1^ Includes any vegetable, including white potatoes, whether baby food or not. Excludes vegetables in mixed dishes. ^2^ Includes commercial and homemade pureed baby-food vegetables. ^3^ Includes non-baby-food dark green, orange, red, starchy, and other vegetables; excludes baby food and white potatoes. ^4^ Includes fried potatoes, mashed potatoes and mixtures, and baked potatoes. ^5^ Includes French fries and any other kind of fried potatoes.

**Table 6:** Percent consuming and grams per capita consumed of meat and other protein food.

Food	Percent Consuming				Grams Per Capita			
	2002	2008	2016	*p*-Value Linear Trend	2002	2008	2016	*p*-Value Linear Trend
	**6–11.9 months**							
**Any meat/other protein food (excludes cheese and yogurt) ^1^**	23	30	37	**<0.0001**	10	20	19	**<0.0001**
Baby-food meats ^2^	4.5	2.5	4.2	NA	2	1	2	NA
Non baby-food meats ^3^	15	23	25	**<0.0001**	5	16	10	**0.0002**
Other protein sources ^4^	7.7	9.3	16	**<0.0001**	3	3	7	**0.0004**
	**12–23.9 months**							
**Any meat/other protein food (excludes cheese and yogurt) ^1^**	82	81	83	0.6193	59	63	73	**0.0001**
Baby-food meats ^2^	2.7	0.8	0.7	NA	1	<0.5	<0.5	NA
Non baby-food meats ^3^	71	74	70	0.4574	40	40	44	0.1330
Other protein sources ^4^	36	35	46	**0.0001**	18	22	29	**<0.0001**

Bolded *p*-values are statistically significant at a value of *p* < 0.001. NA = *p*-value suppressed because it was based on an insufficient number of observations (fewer than 30 consumers or fewer than 30 non-consumers). ^1^ Excludes meat and other protein foods in mixed dishes. Excludes cheese and yogurt; those are reported in Table 2 with dairy products. ^2^ Includes commercial and homemade pureed baby-food meats. ^3^ Includes beef; chicken or turkey; fish or shellfish; hotdogs, sausages, bacon, cold cuts; pork/ham; lamb; goat; game; and organ meats. ^4^ Includes dried beans and legumes; eggs; vegetarian meat substitutes; nuts, nut butters, and seeds; excludes cheese and yogurt; those are reported in Table 2 with dairy products.

**Table 7:** Percent consuming and grams per capita consumed of sweets, sugar-sweetened beverages, and savory snacks.

Food	Percent Consuming				Grams Per Capita			
	2002	2008	2016	*p*-Value Linear Trend	2002	2008	2016	*p*-Value Linear Trend
	**6–11.9 months**							
**All sweets and sugar-sweetened beverages**	44	30	33	**0.0001**	35	26	16	**<0.0001**
**All sweets ^1^**	41	27	29	**<0.0001**	23	13	6	**<0.0001**
**Sugar-sweetened beverages ^2^**	8.2	4.5	7.8	NA	11	12	10	NA
Soft drinks	0.6	0.9	0.4	NA	1	2	<0.5	NA
Fruit-flavored drinks	6.7	3.3	6.8	NA	8	11	9	NA
Sports drinks	0.3	—	0.3	NA	1	—	<0.5	NA
**Savory snacks** ^3^	2.2	0.9	4.1	NA	<0.5	<0.5	1	NA
	**12–23.9 months**							
**All sweets and sugar-sweetened beverages**	86	79	76	**<0.0001**	157	110	93	**<0.0001**
**All sweets ^1^**	80	71	68	**<0.0001**	43	39	30	**<0.0001**
**Sugar-sweetened beverages ^2^**	36	30	25	**<0.0001**	114	72	63	**<0.0001**
Soft drinks	5.3	3.9	1.9	NA	11	7	4	NA
Fruit-flavored drinks	30	22	21	**0.0003**	91	54	52	**0.0002**
Sports drinks	1.3	1.5	1.1	NA	3	3	3	NA
**Savory snacks** ^3^	21	18	18	0.1866	3	3	3	0.3588

Bolded *p*-values are statistically significant at a value of *p* < 0.001. NA = *p*-value suppressed because it was based on an insufficient number of observations (fewer than 30 consumers or fewer than 30 non-consumers). ^1^ Includes sweet baked goods, cereal and nutrition bars, candy, ice cream and other frozen desserts, jellies and jams, milk flavorings, and baby-food desserts and cookies. ^2^ Includes soft drinks, fruit-flavored drinks, tea and coffee, and sports drinks. Excludes 100% fruit juice, which is reported in Table 4 with fruits and 100% fruit juices. ^3^ Includes chips, corn chips, popcorn, snack mix, and puffs (non-baby food).

## Supplementary Materials

The following are available online at https://www.mdpi.com/2072-6643/11/11/2807/s1, Table S1: Grams per consumer for all food and beverage categories among 6–11.9-month-olds and 12–23.9-month-olds across FITS 2002, 2008, and 2016.

## References

[1] Mennella J.A. (2014). Ontogeny of taste preferences: Basic biology and implications for health. Am. J. Clin. Nutr..

[2] Mennella J.A., Reiter A.R., Daniels L.M. (2016). Vegetable and Fruit Acceptance during Infancy: Impact of Ontogeny, Genetics, and Early Experiences. Adv. Nutr..

[3] Rose C.M., Savage J.S., Birch L.L. (2016). Patterns of early dietary exposures have implications for maternal and child weight outcomes. Obesity.

[4] Lioret S., McNaughton S.A., Spence A.C., Crawford D., Campbell K.J. (2013). Tracking of dietary intakes in early childhood: The Melbourne InFANT Program. Eur. J. Clin. Nutr..

[5] Oddy W.H., Mori T.A., Huang R.C., Marsh J.A., Pennell C.E., Chivers P.T., Hands B.P., Jacoby P., Rzehak P., Koletzko B.V. (2014). Early infant feeding and adiposity risk: From infancy to adulthood. Ann. Nutr. Metab..

[6] Kaikkonen J.E., Mikkila V., Magnussen C.G., Juonala M., Viikari J.S., Raitakari O.T. (2013). Does childhood nutrition influence adult cardiovascular disease risk?—Insights from the Young Finns Study. Ann. Med..

[7] Miles G., Siega-Riz A.M. (2017). Trends in Food and Beverage Consumption Among Infants and Toddlers: 2005–2012. Pediatrics.

[8] Fulgoni V.L., Quann E.E. (2012). National trends in beverage consumption in children from birth to 5 years: Analysis of NHANES across three decades. Nutr. J..

[9] Eldridge A.L., Catellier D.J., Hampton J.C., Dwyer J.T., Bailey R.L. (2019). Trends in Mean Nutrient Intakes of US Infants, Toddlers, and Young Children from 3 Feeding Infants and Toddlers Studies (FITS). J. Nutr..

[10] Devaney B., Kalb L., Briefel R., Zavitsky-Novak T., Clusen N., Ziegler P. (2004). Feeding infants and toddlers study: Overview of the study design. J. Am. Diet. Assoc..

[11] Briefel R.R., Kalb L.M., Condon E., Deming D.M., Clusen N.A., Fox M.K., Harnack L., Gemmill E., Stevens M., Reidy K.C. (2010). The Feeding Infants and Toddlers Study 2008: Study design and methods. J. Am. Diet. Assoc..

[12] Anater A.S., Catellier D.J., Levine B.A., Krotki K.P., Jacquier E.F., Eldridge A.L., Bronstein K.E., Harnack L.J., Lorenzana Peasley J.M., Lutes A.C. (2018). The Feeding Infants and Toddlers Study (FITS) 2016: Study Design and Methods. J. Nutr..

[13] Dewey K.G., Finley D.A., Lonnerdal B. (1984). Breast milk volume and composition during late lactation (7–20 months). J. Pediatr. Gastroenterol. Nutr..

[14] Dewey K.G., Lonnerdal B. (1983). Milk and nutrient intake of breast-fed infants from 1 to 6 months: Relation to growth and fatness. J. Pediatr. Gastroenterol. Nutr..

[15] Kent J.C., Mitoulas L., Cox D.B., Owens R.A., Hartmann P.E. (1999). Breast volume and milk production during extended lactation in women. Exp. Physiol..

[16] Ahluwalia N., Herrick K.A., Rossen L.M., Rhodes D., Kit B., Moshfegh A., Dodd K.W. (2016). Usual nutrient intakes of US infants and toddlers generally meet or exceed Dietary Reference Intakes: Findings from NHANES 2009–2012. Am. J. Clin. Nutr..

[17] Grimes C., Szymlek-Gay E., Nicklas T. (2017). Beverage consumption among U.S. children aged 0–24 months: National Health and Nutrition Examination Survey (NHANES). Nutrients.

[18] Kay M., Welker E., Jacquier E., Story M. (2018). Beverage consumption patterns among infants and young children (0–47.9 months): Data from the Feeding Infants and Toddlers Study, 2016. Nutrients.

[19] Benjamin D.J., Berger J.O., Johannesson M., Nosek B.A., Wagenmakers E.J., Berk R., Bollen K.A., Brembs B., Brown L., Camerer C. (2018). Redefine statistical significance. Nat. Hum. Behav..

[20] American Academy of Pediatrics Committee on Nutrition (2014). Pediatric Nutrition: Policy of the American Academy of Pediatrics.

[21] Herrick K.A., Rossen L.M., Kit B.K., Wang C.Y., Ogden C.L. (2016). Trends in Breastfeeding Initiation and Duration by Birth Weight Among US Children, 1999–2012. JAMA Pediatr..

[22] Gurley-Calvez T., Bullinger L., Kapinos K.A. (2018). Effect of the Affordable Care Act on Breastfeeding Outcomes. Am. J. Public Health.

[23] Centers for Disease Control and Prevention Breastfeeding Report Card, United States 2018. https://www.cdc.gov/breastfeeding/pdf/2018breastfeedingreportcard.pdf.

[24] Harris J.L., Fleming-Melici F., Frazier W., Haraghey K., Kalnova S., Romo-Palafox M. (2016). Baby Food FACTS. Nutrition and Marketing of Baby and Toddler Food and Drinks.

[25] Pérez-Escamilla R., Segura-Pérez S., Lott M. (2017). Feeding Guidelines for Infants and Young Toddlers: A Responsive Parenting Approach.

[26] Williams E., Hooper B., Spiro A., Stanner S. (2015). The contribution of yogurt to nutrient intakes across the life course. Nutr. Bull..

[27] Lythgoe A., Roberts C., Madden A.M., Rennie K.L. (2013). Marketing foods to children: A comparison of nutrient content between children’s and non-children’s products. Public Health Nutr..

[28] Tester J.M., Leung C.W., Leak T.M., Laraia B.A. (2017). Recent Uptrend in Whole-Grain Intake Is Absent for Low-Income Adolescents, National Health and Nutrition Examination Survey, 2005–2012. Prev. Chronic. Dis..

[29] Albertson A.M., Reicks M., Joshi N., Gugger C.K. (2016). Whole grain consumption trends and associations with body weight measures in the United States: Results from the cross sectional National Health and Nutrition Examination Survey 2001–2012. Nutr. J..

[30] McGill C.R., Fulgoni V.L., Devareddy L. (2015). Ten-year trends in fiber and whole grain intakes and food sources for the United States population: National Health and Nutrition Examination Survey 2001–2010. Nutrients.

[31] Bailey R.L., Catellier D.J., Jun S., Dwyer J.T., Jacquier E.F., Anater A.S., Eldridge A.L. (2018). Total Usual Nutrient Intakes of US Children (Under 48 Months): Findings from the Feeding Infants and Toddlers Study (FITS) 2016. J. Nutr..

[32] Finn K., Callen C., Bhatia J., Reidy K., Bechard L.J., Carvalho R. (2017). Importance of Dietary Sources of Iron in Infants and Toddlers: Lessons from the FITS Study. Nutrients.

[33] Roess A.A., Jacquier E.F., Catellier D.J., Carvalho R., Lutes A.C., Anater A.S., Dietz W.H. (2018). Food Consumption Patterns of Infants and Toddlers: Findings from the Feeding Infants and Toddlers Study (FITS) 2016. J. Nutr..

[34] Welker E.B., Jacquier E.F., Catellier D.J., Anater A.S., Story M.T. (2018). Room for Improvement Remains in Food Consumption Patterns of Young Children Aged 2–4 Years. J. Nutr..

[35] American Academy of Pediatrics AAP Offers Advice for Parents Concerned about Arsenic in Food. https://www.aap.org/en-us/about-the-aap/aap-press-room/pages/AAP-Offers-Advice-For-Parents-Concerned-About-Arsenic-in-Food.aspx.

[36] Brown A., Jones S.W., Rowan H. (2017). Baby-Led Weaning: The Evidence to Date. Curr. Nutr. Rep..

[37] Grimm K.A., Kim S.A., Yaroch A.L., Scanlon K.S. (2014). Fruit and vegetable intake during infancy and early childhood. Pediatrics.

[38] Kim S.A., Moore L.V., Galuska D., Wright A.P., Harris D., Grummer-Strawn L.M., Merlo C.L., Nihiser A.J., Rhodes D.G. (2014). Vital signs: Fruit and vegetable intake among children—United States, 2003–2010. Morb. Mortal. Wkly Rep..

[39] Fisher J.O., Dwyer J.T. (2016). Next Steps for Science and Policy on Promoting Vegetable Consumption among US Infants and Young Children. Adv. Nutr..

[40] Heyman M.B., Abrams S.A. (2017). Fruit Juice in Infants, Children, and Adolescents: Current Recommendations. Pediatrics.

[41] Sonneville K.R., Long M.W., Rifas-Shiman S.L., Kleinman K., Gillman M.W., Taveras E.M. (2015). Juice and water intake in infancy and later beverage intake and adiposity: Could juice be a gateway drink?. Obesity.

[42] Auerbach B.J., Wolf F.M., Hikida A., Vallila-Buchman P., Littman A., Thompson D., Louden D., Taber D.R., Krieger J. (2017). Fruit Juice and Change in BMI: A Meta-analysis. Pediatrics.

[43] Auerbach B.J., Dibey S., Vallila-Buchman P., Kratz M., Krieger J. (2018). Review of 100% Fruit Juice and Chronic Health Conditions: Implications for Sugar-Sweetened Beverage Policy. Adv. Nutr..

[44] Beck A.L., Patel A., Madsen K. (2013). Trends in sugar-sweetened beverage and 100% fruit juice consumption among California children. Acad. Pediatr..

[45] USDA Food and Nutrition Service (2016). Child and Adult Care Food Program: Meal Pattern Revisions Related to the Healthy, Hunger-Free Kids Act of 2010. Fed. Regist..

[46] Vos M.B., Kaar J.L., Welsh J.A., Van Horn L.V., Feig D.I., Anderson C.A.M., Patel M.J., Cruz Munos J., Krebs N.F., Xanthakos S.A. (2017). Added Sugars and Cardiovascular Disease Risk in Children: A Scientific Statement From the American Heart Association. Circulation.

[47] Bleich S.N., Vercammen K.A., Koma J.W., Li Z. (2018). Trends in Beverage Consumption Among Children and Adults, 2003–2014. Obesity.

[48] Mendez M.A., Miles D.R., Poti J.M., Sotres-Alvarez D., Popkin B.M. (2019). Persistent disparities over time in the distribution of sugar-sweetened beverage intake among children in the United States. Am. J. Clin. Nutr..

[49] Dietz W.H. (2019). We Need a New Approach to Prevent Obesity in Low-Income Minority Populations. Pediatrics.

