# Supplementary material for: Trends in Food Consumption Patterns of US Infants and Toddlers from Feeding Infants and Toddlers Studies (FITS) in 2002, 2008, 2016
Source: Nutrients. 2019 Nov 17;11(11):2807. doi: 10.3390/nu11112807 (PMC6893614; doi:10.3390/nu11112807)
Supplement: Supplementary file 1 [file nutrients-11-02807-s001.pdf]

Supplementary Materials for

Trends in Food Consumption Patterns of US Infants and Toddlers from Feeding Infants and Toddlers Studies (FITS) in 2002, 2008, 2016

Duffy, Kay, Jacquier, Catellier, Hampton, Anater, and Story

Table S1. Grams per consumer for all food and beverage categories among 6-11.9 month olds and 12-23.9 month olds across FITS 2002, 2008, and 2016.

	6-11.9 mos				12-23.9 mos			
**Food**	**2002**	**2008**	**2016**	**Linear Trend**	**2002**	**2008**	**2016**	**Linear Trend**
**All Milk and Milk Products[1]**	727	734	810	NA	613	531	513	NA
Breast Milk	585	574	565	0.2367	436	283	320	0.0092
Formula	670	720	818	**<0.0001**	468	503	501	0.5231
Milk (any)[2]	395	292	360	0.7201	559	489	461	**<0.0001**
Cow's milk[3]	386	294	364	0.8634	555	488	464	**<0.0001**
Whole milk[4]	420	327	476	0.5264	536	489	468	**0.0006**
Reduced fat (2%) milk[4]	314	230	220	NA	519	405	360	**0.0001**
Low- or nonfat milk[4]	57	86	78	NA	361	397	297	0.2184
Flavored milk	141	46	146	NA	312	283	239	0.1613
Toddler milk	56	missing	183	NA	110	708	141	NA
**Cheese**	22	20	19	0.3293	31	30	26	0.0385
**Yogurt**	89	101	95	0.5672	117	113	119	0.6586
**All grain[1]**	62	58	50	0.0033	91	89	99	0.0943
Bread, rolls, biscuits, bagels, & tortillas	20	27	20	0.8254	28	31	36	**<0.0001**
Bread	22	24	20	0.6531	25	29	33	**<0.0001**
Crackers, pretzels, & rice cakes	10	12	12	0.2039	15	17	21	**0.0001**
Crackers	10	11	12	0.2634	14	17	20	**0.0001**
Pancakes, waffles, & French toast	29	29	30	NA	52	42	53	0.6272
Pancakes	31	25	33	NA	59	36	57	0.8198
Pasta & rice	48	79	51	0.9153	79	68	80	0.7720
Pasta	60	40	51	NA	81	65	81	0.6625
Infant cereal[5]	54	36	41	0.0020	46	42	58	0.1685
Family cereal[6]	22	56	39	0.0060	44	52	61	0.0020
Baby finger foods[7]	7	6	5	0.0145	9	13	7	0.0099
**Whole grains (any)[8]**	—	46	43	0.7506	—	52	73	**0.0007**
**Any Fruit[1] or 100% Fruit Juice**	195	154	168	0.0039	301	283	267	0.0044
100% juice[9]	150	126	140	0.4285	260	253	219	0.0030
All fruits (excluding 100% fruit juice)[10]	129	119	126	0.5730	149	148	166	0.0176
Baby food fruit[11]	124	113	120	0.4569	120	119	120	0.9272
**Any vegetable[1, 12]**	103	111	104	0.9107	87	90	88	0.8760
Baby-food vegetables[13]	113	119	113	0.9565	121	136	103	0.1227
Non baby-food vegetables[14]	56	76	57	0.5375	62	66	65	0.4690
White potatoes[15]	40	59	50	0.1689	56	59	60	0.4266
Fried potatoes[16]	27	22	23	NA	41	33	40	0.8158
**Any meat or other protein food (excludes cheese & yogurt)[1]**	45	66	50	0.9356	72	77	88	**<0.0001**
Baby-food meats[17]	54	55	48	NA	47	52	63	NA
Non baby-food meats[18]	33	65	39	0.8832	56	55	63	0.0259
Other protein sources[19]	42	30	40	0.8465	49	62	62	0.0041
**Any sweets or sugar-sweetened beverages**	78	86	48	**0.0001**	180	138	121	**<0.0001**

All sweets[20]	57	49	20	**<0.0001**	53	54	43	0.0065
Sugar-sweetened beverages[21]	136	269	134	NA	291	222	221	0.0023
Soft drinks	40	109	1	NA	132	112	97	NA
Fruit-flavored drinks	125	320	139	NA	288	228	226	0.0164
Sports' drinks	299	—	153	NA	169	167	170	NA
**Savory Snacks**[22]	10	5	15	NA	14	15	19	0.0368

NA = p-value suppressed because it was based on an insufficient number of observations (fewer than 30 consumers or fewer than 30 non-consumers).

— = category not reported for that FITS year.

[1] Excludes foods in mixed dishes.

[2] Includes all milks except breastmilk and infant formula.

[3] Includes all fat levels, as well as flavored, unflavored, or powdered.

[4] Includes only unflavored cow's milk of specified fat level, excludes flavored and powdered.

[5] Includes any kind of baby-food cereal, regardless of grain (i.e., rice, oat, quinoa, wheat, multigrain, or unknown grain).

[6] Includes any ready-to-eat or hot cereal that is not infant cereal.

[7] Includes pretzels, crackers, rice cakes, and baby-food puffs.

[8] Includes products that are ≥50% whole grain. Whole grain intake was not assessed in 2002.

[9] Includes any 100% fruit juice regardless of whether it is specifically labeled for babies or not. Beverages that are <100% fruit juice are included in sugar sweetened beverages.

[10] Includes commercial and homemade pureed baby-food fruit and non-baby-food fruit; excludes 100% juice.

[11] Includes commercial and homemade pureed baby-food fruit, excludes 100% juice.

[12] Includes any vegetable, including white potatoes, whether baby food or not.

[13] Includes commercial and homemade pureed baby-food vegetables.

[14] Includes non-baby-food dark green, orange, red, starchy, and other vegetables; excludes baby food and white potatoes.

[15] Includes fried potatoes, mashed potatoes and mixtures, and baked potatoes.

[16] Includes French fries and any other kind of fried potatoes.

[17] Includes commercial and homemade pureed baby-food meats.

[18] Includes beef; chicken or turkey; fish or shellfish; hotdogs, sausages, bacon, cold cuts; pork/ham; lamb; goat; game; and organ meats.

[19] Includes dried beans and legumes; eggs; vegetarian meat substitutes; nuts, nut butters, and seeds; excludes cheese and yogurt; those are reported in Table 2 with dairy products.

[20] Includes sweet baked goods, cereal and nutrition bars, candy, ice cream and other frozen desserts, jellies and jams, milk flavorings, and baby-food desserts and cookies.

[21] Includes soft drinks, fruit-flavored drinks, tea and coffee, and sports drinks. Excludes 100% fruit juice, which is reported in Table 4 with fruits and 100% fruit juices.

[22] Includes chips, corn chips, popcorn, snack mix, and puffs (non-babyfood).
